# Genetic linkage analysis of longitudinal hypertension phenotypes using three summary measures

**DOI:** 10.1186/1471-2156-4-S1-S24

**Published:** 2003-12-31

**Authors:** Shaoqi Rao, Lin Li, Xia Li, Kathy L Moser, Zheng Guo, Gongqing Shen, Ruth Cannata, Erich Zirzow, Eric J Topol, Qing Wang

**Affiliations:** 1 Center for Cardiovascular Genetics, Department of Cardiovascular Medicine, The Cleveland Clinic Foundation, 9500 Euclid Avenue, Cleveland, Ohio, USA; 2 Department of Molecular Cardiology, Lerner Research Institute, The Cleveland Clinic Foundation, 9500 Euclid Avenue, Cleveland, Ohio, USA; 3 Department of Biomedical Engineering, Biomathematics and Bioinformatics, Harbin Medical University, Harbin, China; 4 Department of Medicine, Institute of Human Genetics, University of Minnesota, Minnesota, USA

## Abstract

**Background:**

Longitudinal data often have multiple (repeated) measures recorded along a time trajectory. For example, the two cohorts from the Framingham Heart Study (GAW13 Problem 1) contain 21 and 5 repeated measures for hypertension phenotypes as well as epidemiological risk factors, respectively. Direct modelling of a large number of serially and biologically correlated traits in the context of linkage analysis can be prohibitively complex. Alternatively, we may consider using univariate transformation for linkage analysis of longitudinal repeated measures.

**Results:**

We evaluated the utility of three conventional summary measures (mean, slope, and principal components) for genetic linkage analysis of longitudinal phenotypes by analyzing the chromosome 10 data of the Framingham Heart Study. Except for the temporal slope, all of the summary methods and the multivariate analysis identified the previously reported region, marker GATA64A09, for systolic blood pressure or high blood pressure. Further analysis revealed that this region may harbor gene(s) affecting human blood pressure at multiple stages of life.

**Conclusion:**

We conclude that mean and principal components are feasible alternatives for genetic linkage analysis of longitudinal phenotypes, but the slope might have a separate genetic basis from that of the original longitudinal phenotypes.

## Background

The Genetic Analysis Workshop 13 (GAW13) for longitudinal hypertension phenotypes, provided by the Framingham Heart Study group [1], is a valuable forum for evaluating existing statistical methodologies and novel approaches for analyzing the data on temporal repeated measures. Together with spatial repetition, longitudinal multiple measurements are the most frequently encountered data structure suitable for repeatability modelling. Repeatability modelling and analysis have a long history [2], and have received renewed attention recently, with development of more sophisticated mixed linear models [3-6]. However, statistical methods for linkage analysis of longitudinal medical phenotypes are in their infancy, partially due to the fact that a large number of temporal repeated measures are often obtained for such data as that from the Framingham Heart Study. Direct multivariate modelling of these data can be prohibitively complex. Alternatively, we may consider transforming the multivariate linkage analysis into univariate analysis through some summary measures such as the arithmetic mean and temporal slope that are commonly used by biostatisticians in longitudinal data analysis, or the derived statistically uncorrelated principal components [7,8]. The purpose of this study was to evaluate the utility of the three data transformation methods for genetic linkage analysis of longitudinal phenotypes by analyzing the chromosome 10 data from the Framingham Heart Study.

## Methods

### Linkage analysis for individual repeated measures of hypertension phenotypes

Framingham Heart Study data sets for GAW13 Problem 1 contain up to 21 and 5 longitudinal systolic blood pressures (SBP) and the derived high blood pressure (HBP, HBP = 1 if SBP ≥ 140 or diastolic BP ≥ 90) measures as well as measures for numerous risk factors or related traits with cardiovascular diseases, respectively. We first considered analyzing the individual longitudinal measures separately. Although linkage analysis of individual repeated measures separately may lose some important loci that presumably have pleiotropic effects on multiple repeated measures, most of the major genes that turn on and off at different temporal stages should be detected via marginal analysis of the individual phenotypes.

A particular characteristic of the data set is that a large proportion of members in the original cohort did not have genotype data although almost the same amount of phenotype information was available as for the offspring cohort. The number of informative sib pairs (about 50, taking up <5% of the total number of informative sib pairs for the offspring cohort) is too small to render a reliable sib pair linkage analysis. Because of this and the significant difficulty in merging the two cohorts, we dropped Cohort 1 from this analysis. To make consistent comparisons with the previously published results [1], we adopted similar strategies for adjusting covariates, but a linear adjustment was applied to antihypertensive treatment. Namely, prior to linkage analysis, the residuals after removing effects of sex, age, body mass index (BMI, calculated as the weight in kilograms divided by the square of height in meters (kg/m^2^)) and antihypertensive treatment (coded as 1 if the participant took medication and 0 otherwise) were obtained. Then, the residuals were analyzed using the new Haseman-Elston regression [9]. SAS general linear model analysis indicated that all the factors but sex were important contributors (*P *< 0.0001) for both the five longitudinal phenotypes of SBP (SBP1-SBP5) and HBP (HBP1-HBP5) in the offspring cohort, respectively.

### Linkage analysis of arithmetic means of multiple temporal measures

We essentially repeated the analysis of Levy et al. [1]. First, within-subject mean SBP and HBP as well as mean age, BMI, and antihypertensive treatment (mean number of treatments) were calculated. Then, a general linear model was used to adjust for sex, age, BMI, and antihypertensive treatment, separately for each cohort. Next, the residuals for both cohorts were merged and were used in the sib-pair regression-based linkage analysis. Again, all the factors but sex were important contributors (*P *< 0.0001) for the mean summaries of longitudinal SBP and HBP phenotypes for both cohorts, respectively.

### Linkage analysis of temporal slopes for systolic blood pressure

The subject-specific temporal slopes were obtained separately for each cohort and for each subject, by fitting a regression of the continuous SBP on the actual age at which the item had been measured. The estimated slopes were then adjusted for sex, mean BMI, and antihypertensive treatment. Next, the adjusted slopes for the two cohorts were merged and were used in the following linkage analysis. In contrast with the above two kinds of longitudinal phenotypes, sex was an important factor (*P *< 0.0001) for the temporal slope for the offspring cohort, and the importance of BMI and antihypertensive treatment (in terms of *P *values) was decreased.

### Linkage analysis of principal components

For the same reasons as in the first approach, we removed the Cohort 1 from this analysis. To make consistent comparisons among these longitudinal measures, we first adjusted for the effects of the four covariates (sex, age, BMI, and antihypertensive treatment), as was done in the first approach. All five longitudinal SBPs and HBPs were standardized before obtaining the principal components. For the purpose of obtaining the principal components, all individuals in this analysis were considered to be independent, and only those individuals (*n *= 1119, 1106, for SBP and HBP, respectively) with measures on all five time points were included. The eigenvalues and coefficients (loading matrix) are shown in Table 1. The first three principal components account for 84% and 75% of the total variation for SBP and HBP, respectively. The computed principal components were then subject to linkage analysis, and were evaluated univariately and multivariately as described previously [7]. Note that for both traits, the coefficients for the five longitudinal phenotypes are roughly equal in principal component 1 (PRIN1), suggesting that linkage results for this component would be similar to those for the mean measure with an equal weight (0.20) for all five longitudinal phenotypes. Furthermore, the remaining principal components are easily recognized as approximately the linear (straight line), quadratic (a parabola), cubic (the third degree polynomial), and quartic trends (the fourth degree polynomial) of SBP and HBP along the longitudinal trajectory.

## Results

### Evaluation of the three summary methods in terms of gene localization and statistical significance

**Figure 1:**
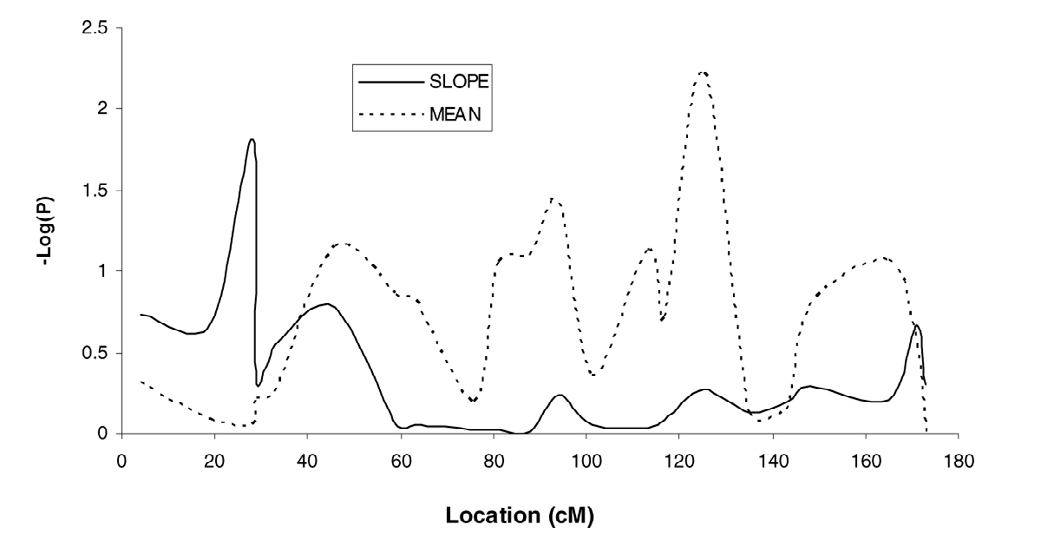
**Linkage profiles for two longitudinal summary measures **Lines depict the -log_10 _(*P*-value) along chromosome 10. The dashed line indicates the arithmetic mean and the solid line indicates the temporal slope of longitudinal systolic blood pressures. The results were obtained by new Haseman-Elston regression using SAGE package.

The summary of linked regions (*P *< 0.01) for SBP and HBP identified using different longitudinal measures is shown in Table 2. It is interesting to note that except for linkage analysis of individually repeated HBP measures and temporal slope, all methods identified marker GATA64A09 at 125 cM on the chromosome 10 as a marker showing significant linkage, which was also reported previously [1]. Further analysis revealed that this region may harbor gene(s) affecting blood pressure at multiple temporal stages (SBP1, SBP2, and SBP4), and this may be why it was detected using two summary measures (mean and the first principal component). Through analysis of individual repeated measures, we identified a novel region (marker GATA70E11 at 46 cM on chromosome 10) that had a specific effect on blood pressure (and hypertension) at the early stage of life. Contrary to our early finding [7], this study indicates that standard principal component analysis indeed decomposed the total variance with respect to genetic components. Nevertheless, our previous speculation that statistical efficiency (in terms of *P *values) for linkage analysis of principal components is higher than that for individual measures [8] is supported by this study. The fact that no regions had been detected to be significantly linked to the temporal slope of SBP and that the obvious differences between linkage profiles for the temporal slope and for the arithmetic mean of longitudinal SBPs exist (Figure 1) indicate that the temporal slope might have a separate genetic basis from that for the original longitudinal phenotypes and other summary measures.

### (Multivariate) linkage analysis via principal components

**Figure 2:**
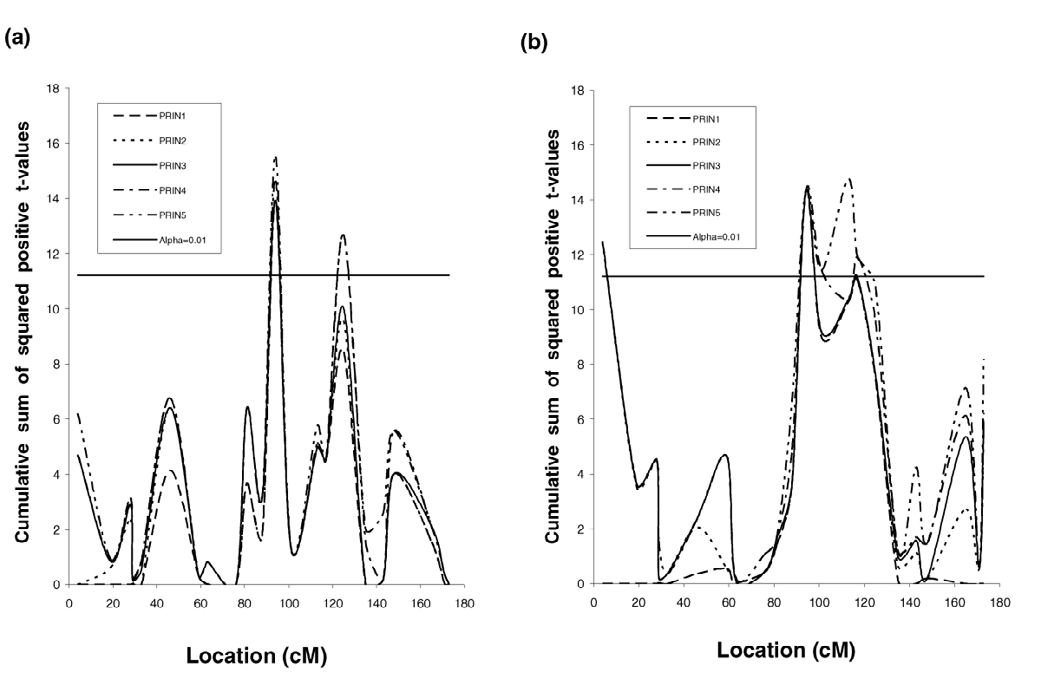
**Cumulative *t*^2 ^statistics for five principal components (PRIN1-PRIN5) of two longitudinal phenotypes **Each line depicts the sum of *t*^2 ^values (for *t *> 0) for all of principal components up to and including the i^th ^for systolic blood pressure (a) and hypertension (b).

To test the joint effects of a putative gene(s) on multiple longitudinal SBPs and HBPs, we applied a multivariate statistical testing procedure to the derived principal components. We set the negative estimates of the Haseman-Elston regression slope to zero to account for the one-sided nature of the *t*-tests. We defined the overall multivariate statistic to be the sum of those *t*^2 ^statistics for which t > 0. The multivariate critical values at α = 0.05, 0.01, 0.005, and 0.001 are 7.49, 11.20, 12.75, and 16.33, respectively. Figure 2 shows the cumulative *t*^2 ^statistic profiles for the five principal components of longitudinal SBPs and HBPs, respectively. We identified four multivariate linkage regions (*P *< 0.01), two for each trait. The two regions for SBP correspond to markers GATA87G01 (*P *= 0.0014) and GATA64A09 (*P *= 0.0052). One region for HBP spans a 31-cM interval containing five markers (GATA87G01-GATA64A09), with the peak at marker GATA115E01 (*P *= 0.0020) and the second region is at the terminal marker (GATA88F09, *P *= 0.0057).

## Discussion

In this study we have evaluated the utility of three summary measures for genetic linkage analysis using the chromosome 10 data from the Framingham Heart Study. Our study supports the feasibility of mean and principal components as alternative phenotypes for longitudinal measures. The mean summary is analogous to principal components in that both are a linear function of the original traits, but the principal component approach is clearly superior because of its mathematical soundness and the ability to test more complicated genetic hypotheses [8]. The temporal within-subject slope measure is analogous to random regression modelling for genetic analysis of longitudinal varying traits [5]. The limited evidence suggests that the temporal slope might have a separate genetic basis.

We adopted a two-step approach to longitudinal linkage analysis. It has an advantage of simplicity and the resultant summaries are easily understood. Biologically and genetically, mean and slope summaries can be used to study genes varying in the course of life or genes having significant differential effects on hypertension phenotypes over time. The principal component analysis here is essentially the trend analysis in the repeated measures modelling. Not surprisingly, the results for the PRIN1 correspond closely to those for the mean summary. PRIN2, approximately the linear trend, identified two linkage signals for HBP phenotype, but they were not detected (or at least not significantly) with slope. As we expected and as was suggested by this study, the genes that influences trends of higher orders (quadratic, cubic, and quartic trends, corresponding to PRIN3-PRIN5) are difficult to detect. Interestingly, several groups for GAW13 took hierarchical modelling approaches, which can be considered a systematic way to the two-step approach. Under the assumption of homogeneous with-subject variability over time, the two approaches are identical. However, if there is marked heteroscedacity of variance for the summary measures resulted from whatever reasons (for example, differences in the true within-subject variability over time or differences in the number of observation available or their distribution by age), a unified hierarchical analysis of the two steps that takes this into account automatically is desirable.

The multivariate approach used in this study for evaluating the joint actions of gene(s) for hypertension phenotypes was originally proposed to handle multiple disease-related phenotypes [7]. Here, we extend it to the multiple longitudinal temporal measures of basically the same trait, for which the multivariate *P *values might be interpreted differently. If we are willing to accept the notion that multiple longitudinal hypertension phenotypes have the same (or similar) genetic basis, then, the multivariate test reveals which gene(s) were active during the multiple stages of life. The facts that the marker GATA64A09 attained a multivariate significance (*P *< 0.01) for both longitudinal SBP and the derived hypertension and a univariate significance (*P *< 0.01) for SBP at multiple stages of life would strongly support the joint (pseduopleiotropic) effects of the putative gene(s). However, we should point out that the multivariate approach based on principal components was developed to handle pleiotropic effects of a gene and it cannot detect interactions between genes or between genes and environments, for which a sophisticated method such as step-wise discriminant analysis used in our separate GAW13 paper [10] is needed. For example, we suspected that there are gene × gene interactions in a 31-cM interval identified to be significantly linked to HBP by the multivariate testing. Further analysis by stepwise discriminant analysis in the separate study indeed suggests the existence of gene × gene interactions between markers GATA64A09 and GATA115E01.

Selections of covariates and adjustment strategies for this study were made in accordance with the previously published paper [1]. They are neither necessarily the best nor the most efficient. In addition, there are uncertainties in the adjusted values based on the linear model that are not accounted for in the linkage analysis, so the true linkage signals from the subsequent linkage analysis could be either inflated or missed. To clarify these issues, a large simulation study such as GAW13 Problem 2 should be undertaken, which is beyond the scope of this work.

## Conclusions

The linkage analysis using three summary measures (mean, slope, and principal components) supports the utility of univariate transformation from multiple longitudinal measures as an alternative for direct multivariate modelling, but interpretations of different summary measures in the context of genetics are different.

## Figures and Tables

**Table 1:** Principal component analysis for five SBP values (SBP1-SBP5) and hypertension phenotypes (HBP1-HBP5), respectively^A^

Principal component	Eigenvalue	Proportion of variance	Cumulative proportion of variance	Coefficient				
SBP				SBP1	SBP2	SBP3	SBP4	SBP5
				
PRIN1	2.862	0.573	0.573	0.36	0.46	0.49	0.47	0.43
PRIN2	0.767	0.153	0.726	0.83	0.20	-0.14	-0.35	-0.37
PRIN3	0.565	0.113	0.839	0.36	-0.52	-0.33	-0.07	0.70
PRIN4	0.436	0.087	0.926	-0.24	0.62	-0.24	-0.56	0.42
PRIN5	0.368	0.074	1.000	0.00	0.29	-0.76	0.58	-0.90
HBP				HBP1	HBP2	HBP3	HBP4	HBP5
				
PRIN1	1.991	0.398	0.398	0.33	0.44	0.52	0.51	0.41
PRIN2	0.964	0.193	0.591	0.67	0.42	-0.08	-0.27	-0.54
PRIN3	0.813	0.163	0.754	0.64	-0.50	-0.38	0.05	0.44
PRIN4	0.661	0.132	0.886	-0.12	0.52	-0.28	-0.55	0.58
PRIN5	0.571	0.114	1.000	0.13	-0.33	0.71	-0.60	0.10

^A^Prior to obtaining the principal components, all five SBPs and HBPs were adjusted for the effects of four covariates (sex, age, body mass index, and antihypertensive treatment) and were standardized.

**Table 2:** Summary of linked regions (*P *< 0.01) to SBP and HBP identified using different longitudinal measures

Method	Traits	Marker	Position (cM)	*P*-Value
Individual time point (Cohort 2)	SBP	GATA70E11 (SBP1)	46	0.00005
		GATA64A09 (SBP1)	125	0.00782
		GATA64A09 (SBP2)	125	0.00571
		GATA64A09 (SBP4)	125	0.00279
	HBP	GATA70E11 (HBP1)	46	0.00691
		GGAA23C05 (HBP1)	165	0.00767

Mean	SBP	GATA64A09 (Adjustment)	125	0.00599
		GATA64A09 (No adjustment)^A^	125	0.00492
	HBP	GATA87G01 (Adjustment)	94	0.00196
		GGAA2F11 (No adjustment)	117	0.00407
		GATA64A09 (No adjustment)	125	0.00618

Slope	SBP	Null	-	-

Principal component (Cohort 2)	SBP	GATA87G01 (PRIN1)	94	0.00010
		GATA64A09 (PRIN1)	125	0.00190
	HBP	GATA87G01 (PRIN1)	94	0.00008
		GGAT1A4 (PRIN1)	101	0.00140
		GATA115E01 (PRIN1)	113	0.00070
		GGAA2F11 (PRIN1)	117	0.00046
		GATA64A09 (PRIN1)	125	0.00317
		GATA88F09 (PRIN2)	4	0.00022
		Mfd187 (PRIN2)	173	0.00915

^A^No covariate adjustment was made.
